# A multiple kernel density clustering algorithm for incomplete datasets in bioinformatics

**DOI:** 10.1186/s12918-018-0630-6

**Published:** 2018-11-22

**Authors:** Longlong Liao, Kenli Li, Keqin Li, Canqun Yang, Qi Tian

**Affiliations:** 1 College of Computer, National University of Defense Technology, Sanyi Road, Changsha, China; 2 State Key Laboratory of High Performance Computing, Sanyi Road, Changsha, China; 3 College of Information Science and Engineering, Hunan University, Lushan Road, Changsha, China; 4 Department of Computer Science, State University of New York, Road, New Paltz, USA; 5 Department of Computer Science, University of Texas at San Antonio, Road, San Antonio, USA

**Keywords:** Density clustering, Matrix completion, Unsupervised multiple kernel learning, Dimensionality reduction, Outlier detection

## Abstract

**Background:**

While there are a large number of bioinformatics datasets for clustering, many of them are incomplete, i.e., missing attribute values in some data samples needed by clustering algorithms. A variety of clustering algorithms have been proposed in the past years, but they usually are limited to cluster on the complete dataset. Besides, conventional clustering algorithms cannot obtain a trade-off between accuracy and efficiency of the clustering process since many essential parameters are determined by the human user’s experience.

**Results:**

The paper proposes a Multiple Kernel Density Clustering algorithm for Incomplete datasets called MKDCI. The MKDCI algorithm consists of recovering missing attribute values of input data samples, learning an optimally combined kernel for clustering the input dataset, reducing dimensionality with the optimal kernel based on multiple basis kernels, detecting cluster centroids with the Isolation Forests method, assigning clusters with arbitrary shape and visualizing the results.

**Conclusions:**

Extensive experiments on several well-known clustering datasets in bioinformatics field demonstrate the effectiveness of the proposed MKDCI algorithm. Compared with existing density clustering algorithms and parameter-free clustering algorithms, the proposed MKDCI algorithm tends to automatically produce clusters of better quality on the incomplete dataset in bioinformatics.

## Background

Any non-uniform data contains an underlying structure due to the heterogeneity of the data, the process of identifying this structure in terms of grouping the data samples is called clustering, and the resulting groups are called clusters. The grouping is usually based on the similarity measurements defined for the data samples. Clustering provides a meaningful data analysis method concerning data mining and data classification from large-scale data samples, which is mostly used as an unsupervised learning method in a wide range of areas, for example, bioinformatics, biomedicine and pattern recognition. It aims at finding hidden structure, identifying clusters with similar characteristics in given datasets, and then grouping the similar samples into the same cluster and classify different data samples into different clusters. Thus, over the past years, a number of clustering algorithms have been proposed and improved. The most popular clustering methods include partition-based (e.g., *k*-means [1] and *k*-means ^++^ [2]), density-based (e.g., DBSCAN [3], DENCLUE [4] and CFSFDP [5]), graph-based (e.g., Spectral [6]), and hierarchical (e.g., BIRCH [7] and ROCK [8]) methods.

Most of the proposed clustering algorithms assume that the input dataset is complete during the past few years, they are not applicable directly if the input dataset is incomplete, i.e., attribute values of some elements in the datasets are missing. In reality, many large-scale datasets are incomplete due to various reasons. Thus, it is essential to make the proposed clustering algorithm to work on the incomplete datasets, by recovering missing attribute values of incomplete samples in the input datasets. Besides, compared with other clustering methods, the clusters in the density clustering are the areas with a higher density than their neighbors and a relatively larger dissimilarity from other samples of the given dataset with higher density; they also have an arbitrary shape in the attribute space. However, most of existing density clustering algorithms are effective only when the human users set appropriate parameters, for example, distance threshold, the minimum number of samples to form a cluster, and etc. The performance of clustering results is significantly affected by these input parameters. Human users need to guess them via several exploratory processes that make it more inconvenient.

Traditional multiple kernel learning (MKL) methods are supervised learning since that the kernel learning task requires the class labels of training data samples. Nevertheless, class labels may not always be available in some real-world scenarios beforehand, e.g., an unsupervised learning task such as clustering and dimension reduction. Unsupervised Multiple Kernel Learning (UMKL) is an unsupervised learning method. It does not require class labels of training data as needed in a conventional multiple kernel learning task. Then, it learns an optimal kernel based on multiple predefined basis kernels and an unlabeled dataset [9].

In a previous study, we have proposed a density clustering approach with multiple kernels for high-dimension bioinformatics dataset [10]. However, this initial study did not provide detailed studies for the multiple kernel density clustering approach on incomplete datasets. In this work, we present a Multiple Kernel Density Clustering algorithm for Incomplete datasets in bioinformatics, which is called MKDCI. In the MKDCI method, the incomplete dataset is completed with matrix completion method based on spare self-representation, then the cluster centroids are automatically spotted with the Isolation Forests method, and the clusters with an arbitrary shape are easily obtained by the proposed multiple kernel density clustering method. Differing from existing density clustering algorithms, the MKDCI algorithm functions automatic determination of relative parameters for clustering incomplete datasets, including the optimal value of cut-off distance, the optimal combination of multiple basis kernels, number of clusters and centroids. Besides overcoming the limitation of determining many critical parameters manually during clustering process, the proposed MKDCI algorithm works on the high-dimensional incomplete dataset and obtains clustering results with improved accuracy and stability.

MKDCI clustering algorithm is evaluated by using an extensive set of well-known bioinformatics datasets and widely accepted clustering evaluation metrics that are briefly described in the related section, with reasons why these datasets are used. The excellent quality of the proposed MKDCI algorithm arises from its key features. In particular:

- It recovers the missing attributes values in the input dataset by utilizing matrix completion based on sparse self-representation, instead of directly fills the missing attributes with average value or deletes the data samples with missing attributes from the input dataset.
- It learns an optimal kernel based on multiple predefined basis kernels with a UMKL method, and obtains the optimal value of cut-off distance *d*_*c*_ with entropic affinity, as opposed to adopt the strategy for determining parameter *d*_*c*_ as described in [5].
- It automatically detects cluster centroids of the given dataset by using the Isolation Forests method [11], which is based on the distribution of local density *ρ*_*i*_ of each data sample and its minimum distance *δ*_*i*_ from other data samples with higher density.
- It clusters high-dimensional data samples and visualizes the results efficiently with Multiple Kernel t-Distributed Stochastic Neighbor Embedding (MKt-SNE).

The remaining parts of the paper are organized as follows: In the next section, a brief overview of existing literature about matrix completion, density clustering algorithms and parameter-free clustering algorithms are presented. Then, the proposed MKDCI algorithm is discussed thoroughly, including formal definition of the problem, steps, and mathematical properties. In the final section, the selected bioinformatics clustering datasets and their pre-processing approaches are introduced, the tricks of the MKDCI implementation and quality evaluation metrics, and discusses the extensive experimental evaluation and its results.

## Related work

It is a difficult task to perform clustering on the incomplete datasets in which some data samples contain missing attribute values, but the missing value imputation can be utilized to predict missing attribute values by reasoning from the observed attribute values of other data samples. Consequently, the effectiveness of missing value imputation is dependent on the observed attribute values of other data samples in the incomplete datasets, the imputation of missing attribute values impacts on the clustering performance. To deal with *k*-means clustering on the incomplete datasets, the similarity between two incomplete data samples is measured with the distribution of the incomplete attributes [1]. Collective Kernel Learning [12] collectively completes the kernel matrices of incomplete datasets by inferring hidden sample similarity from multiple incomplete datasets. However, it is limited to deal with multiple incomplete datasets that share common data samples and cover all data samples, i.e., there are no missing data samples in the intersection set of data samples coming from all incomplete datasets.

Matrix completion is to recover an incomplete matrix where part of elements is missing. Linear matrix completion methods assume that the given data come from linear transformations of low dimensional subspace and the data matrix is low-rank. The property of low-rank is utilized to recover the missing elements in the data matrices by minimizing the matrix rank, and the missing elements of a low-rank matrix can be recovered with high probability under the constraints of missing rate, matrix rank, and sampling scheme [13]. Matrix factorization and rank minimization are two classic linear matrix complete methods. For the matrix factorization based matrix completion methods, its main idea is that an *m*×*n* matrix of rank-*r* can be factorized into two smaller matrices of size *m*×*r* and *r*×*n*, where *r*<*min*(*m*,*n*), the missing elements are predicted by finding such pairwise matrices [14]. For rank minimization based matrix completion methods, nuclear-norm is the sum of the singular values of a matrix, and a number of extensions of nuclear-norm are utilized to complete the matrices with missing elements. For example, Schatten *p*-Norm [15] is used to recover incomplete matrices, defined as the *p*-root of the sum of singular values’ *p*-power.

Nuclear-norm is a special case of Schatten *p*-norm when *p*=1. Truncated nuclear-norm [16] refers to the nuclear-norm subtracted by the sum of the largest few singular values, and tends to get the better approximation than nuclear-norm for matrix rank since that the largest few singular elements contain important information and should be preserved. The iteratively reweighted nuclear-norm algorithm [17] is proposed to deal with Schatten *p*-Norm of the low-rank minimization problem, and the evaluation results show that Schatten *p*-Norm outperformed other non-convex non-smooth extensions of rank-minimization. Besides, a spare self-representation based matrix completion method is proposed for predicting missing elements of the incomplete matrices drawn from multiple subspaces [18].

Following the proposal of *k*-means clustering approach, hundreds of new clustering methods have been introduced in literature, especially in the last 20 years many variants of classical clustering problems have been studied, such as partition-based clustering, hierarchical clustering algorithms, graph-based clustering and density-based clustering.

The key of partition-based clustering methods is that they initially partition the dataset into *k* clusters and then iteratively improve the accuracy of clustering by reassigning the data samples to a more appropriate cluster. One of the most widely used clustering algorithms of this kind is *k*-means [1], owing to its efficiency and logical simplicity. The *k*-means algorithm randomly selects *k* samples as initial *k* cluster centroids and assigns the remaining samples to the nearest cluster regarding the distance metric between them and the cluster centroids, such as Euclidean distance and Mahalanobis distance. Then, it iteratively updates the centroids as the new initial cluster centroids and reassigns the remaining samples to the newly computed centroids, until the cluster reassignment no longer changes at each iteration. *k*-means tends to generate approximately equal sized clusters for minimizing intra-cluster distances and has the poor performance when it is used to reproduce clusters for the given dataset with the distribution of complex shape. *k*-means++ [2] improves the performance of *k*-means by optimizing the initial seeding, which reduces the variability of the cluster results by using the distance-based probabilistic approach to selecting the *k* initial centroids. However, most of the partition-based clustering methods have a serious shortcoming that the clustering performance relies heavily on the initial parameter *k*. They tend to obtain a local optimum result rather than a global one.

Hierarchical clustering algorithms can be classified into two main categories: divisive clustering algorithms and agglomerative clustering algorithms. The divisive clustering algorithms start from all samples as one cluster and then recursively divides the cluster into many smaller ones until the expected clusters are produced. Instead, the agglomerative approaches, such as BIRCH [7] and ROCK [8], initial every sample as a cluster and then iteratively merges pairs of clusters till obtaining the expected number of clusters. Unfortunately, they are sensitive to the clustering shape and slower than the partition-based clustering methods.

The graph-based clustering algorithms represent the non-uniform data samples as a graph, where a vertex denotes a data sample, and the weight of an edge denotes the similarity between the two data points connected by the edge. Then a graph cut method is applied to cut the whole graph into several sub-graphs, and each sub-graph is a cluster. Spectral clustering is a widely used graph-based clustering algorithm, and it can be implemented efficiently with standard linear algebra methods [19]. The main shortcoming of graph-based clustering algorithms is the computational bottleneck.

Density-based clustering algorithms find the points with higher density as the cluster centroids over the distribution of data samples [20]. The data samples having the higher density over a region will form a cluster, such as DBSCAN [3], DENCLUE [4] and CFSFDP [5].

DBSCAN algorithm uses the distance of data samples to create a neighboring relation, implies prior information of radius and minimum point number to form a cluster, and it has shown good clustering performance on the arbitrarily shaped distribution of data samples. However, DBSCAN clustering algorithm has two shortcomings: (1) Clustering results heavily depend on the maximum radius of a neighborhood and the minimum number of the data samples contained in this neighborhood. Nevertheless, these two parameters are difficult to be determined by human users. (2) Given the assumption that clusters have similar densities, DBSCAN tends to obtain unintended clustering results on varying densities of datasets. Compared with DBSCAN, DBSCAN-GM [21] method tries to find suitable parameters for DBSCAN, which uses Gaussian Means to find a radial distance and a minimum number of points to form clusters. Hierarchical Density-Based Spatial Clustering (HDBSCAN) [22] forms clusters of different densities with varying epsilon values and is more robust for corresponding parameter selection.

DENCLUE [4] algorithm utilizes the Gaussian kernel density estimation to define clusters and assigns the data samples with the similarity local density maximum to the same cluster. Owing to the hill climbing approach is utilized, it may run unnecessary small steps in the beginning and never exactly converges to the maximum. DENCLUE 2.0 [23] introduces a new hill climbing method for Gaussian kernels, which adjusts the step size automatically at no extra costs, and the procedure converges precisely towards a local maximum by reducing it to a special case of the expectation maximization algorithm. It needs fewer iterations and can be accelerated, but the accuracy of clustering results is decreased.

“Clustering by fast search and find of density peaks (CFSFDP)” [5] is a classic density clustering algorithm, which can generate the clusters regardless of its density distribution and dimensions of data samples. This method has efficient performance since that the whole process of clustering only iterates the data points once, and can correctly recognize clusters regardless of their shape. However, this approach has several limitations as follows: (1) It requires manual determination of a cut-off threshold in the decision graph to determine the density peaks. The cut-off threshold is a cut-off distance used to calculate the local density of each data point. It is set by users with respect to their experience. The choice of the cut-off threshold for the given dataset is usually inefficient and difficult in two special cases. One case is that the data points with lower (or higher) local density and higher (or lower) relative distance are hard to be determined whether they are chosen as the density peaks or not. The other case is that it results in one cluster is erroneously divided into multiple sub-clusters when there is more than one density peak in the same cluster. (2) The clustering results are influenced by kernel functions used in dissimilarity computation, such as Gaussian kernel, Exponential kernel, Truncated kernel, Gravity kernel, etc. (3) The read and write of the input distance matrix of CFSFDP algorithm always exceeds the memory of personal computers for clustering the large-scale dataset.

Kernel clustering algorithms can capture the non-linear structure inherent in various datasets, such as kernel *k*-means and spectral clustering, and usually achieve better clustering performance and identify arbitrarily shaped clusters. Spectral clustering is a weighted variant of kernel *k*-means clustering algorithm. However, the performance of the single kernel methods is largely determined by choice of kernel functions. Unfortunately, the most appropriate kernel function for the target clustering task is often unknown in advance, and it is time-consuming to search exhaustively when the size of the user-defined pool of basis kernels is large [24].

Besides, single kernel methods tend to fail to utilize the heterogeneous features of the datasets fully, but most data samples are represented by multiple groups of features. Therefore, multiple kernel methods are proposed to leverage the different features of the clustering datasets fully. They can learn an appropriate kernel efficiently to make the kernel *k*-means clustering robust and improved in various scenarios [25]. Multiple kernel learning algorithms attempt to optimize the combination kernel by maximizing the centralized kernel alignment between the combined kernel and the ideal kernel [26]. These multiple kernel clustering algorithms belong to supervised kernel learning and require the class labels of training data samples.

Differing from above clustering algorithm, Parameter Free Clustering (PFClust) [27] can automatically cluster data and identify a suitable number of clusters to group them without requiring any parameters to be specified by the human users. It partitions the input dataset into a number of clusters that share some common attributes, such as their minimum expectation value and variance of intra-cluster similarity. However, its performance on clustering high-dimensional datasets is poor.

## Methods

Given an input dataset *X*^*n*×*d*^={*x*_1_,*x*_2_,…,*x*_*n*_} is a set containing *n* data samples, and each data sample has *d* attributes. The high dimensional dataset [28] means that the number of attribute values for each data sample is larger than ten, i.e., *d*>10. By predefining several basis kernel functions, e.g., Gaussian kernel, Exponential kernel, and Laplace kernel, the proposed MKDCI algorithm aims to generate a cluster partition *D*={*D*_1_,*D*_2_,…,*D*_*k*_} with 0<*k*<*n* for the data samples in the input dataset *X*, such that data samples in the same cluster could have larger similarity than others in the different clusters. Thus, the proposed MKDCI algorithm is illustrated in Algorithm 1.


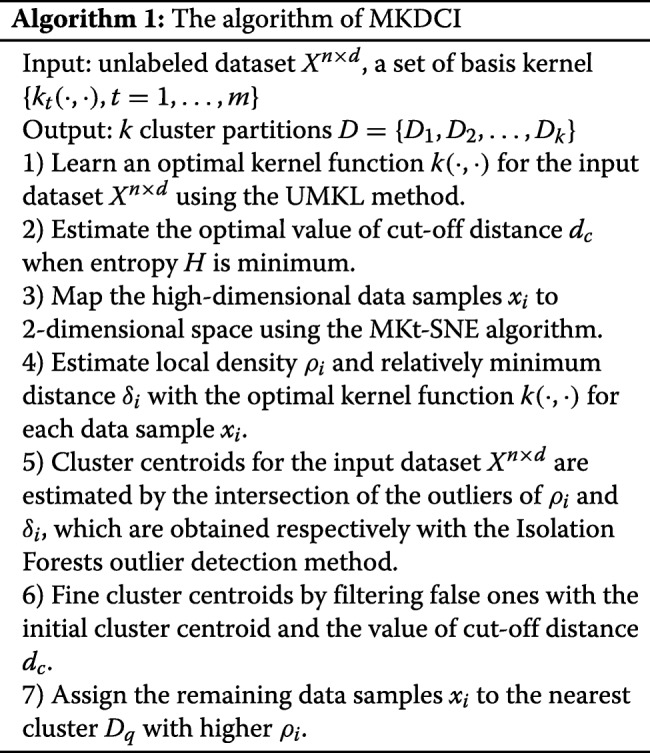


### Completeness of incomplete datasets based on matrix completion

A variety of bioinformatics datasets are naturally organized in matrix form since that the matrix provides a convenient way for storing and analyzing a wide range of bioinformatics data samples. However, a large number of bioinformatics datasets are incomplete in many practical scenarios, in other words, there are missing values in the matrix form of the dataset. The missing values usually raise from failures in data sampling processes. Matrix completion [29] is an effective method to fill the missing elements of an incomplete matrix and recover the entire matrix format of bioinformatics datasets.

Conventional matrix completion approaches are based on rank minimization, they are limited to process the low-rank incomplete matrices, and the data samples are sampled from a single low-dimensional subspace. The approach of completing matrix based on sparse self-representation [18], can recover matrices with following properties: (a) the dimensions of each element in the matrices are unknown; (b) the incomplete matrix is a high-rank or full-rank matrix, and not limited to the low-rank matrix.

Given an incomplete matrix **X**^*n*×*d*^ in which the observed values are {**M**_*i*,*j*_,(*i*,*j*)∈*N*}, each column of matrix **X** can be represented by a linear combination of other columns, matrix completion is to predict the missing values in matrix **X**. Matrix self-representation refers to represent the matrix **X** by itself multiplying a non-identity matrix **S**, i.e., **X**=**XS**, and each element *S*_*i*,*j*_ implies the contribution of the *i*-th column to the *j*-th column of matrix **X**. Since a set of basis vectors of **X** are defined by different subsets of columns of **X**, **S** is not unique, an efficient representation of **X** with a penalized **S** can be computed as follow:

1 $$  \min\limits_{\mathbf{S}}\parallel \mathbf{S} \parallel_{l_{\mathbf{S}}} ~~s.t.~ \mathbf{X}=\mathbf{X} \mathbf{S}  $$

where $\parallel \mathbf {S} \parallel _{l_{\mathbf {S}}}$ denotes a specific regularization operator on **S**. To minimize the representation errors, Eq. (1) is extended to

2 $$  \min\limits_{\mathbf{S}}\parallel \mathbf{S} \parallel_{l_{\mathbf{S}}}+\frac{\partial}{2} \parallel\mathbf{X}-\mathbf{X} \mathbf{S}\parallel_{F}^{2}  $$

where *∂* is a regularization parameter, with a convex $\parallel \mathbf {S} \parallel _{l_{\mathbf {S}}}$ and a specific parameter *∂*, the solutions S of Eqs. (1) and (2) are different. Assuming that the locations of the elements of matrix **X** are *N*, and **X**_*i*,*j*_=**M**_*i*,*j*_,∀(*i*,*j*)∈*N*, the matrix completion can be finished by solving the following optimization problems.

3 $$  \begin{aligned} \min\limits_{\mathbf{X},\mathbf{S}}\parallel \mathbf{S} \parallel_{l_{\mathbf{S}}} ~~ s.t.~ & \mathbf{X} =\mathbf{X} \mathbf{S},\\ & \mathbf{X}_{i,j}=\mathbf{M}_{i,j},\forall (i,j) \in N \end{aligned}  $$

4 $$  \begin{aligned} \min\limits_{\mathbf{X},\mathbf{S}}\parallel \mathbf{S} \parallel_{l_{\mathbf{S}}}+\frac{\partial}{2} & \parallel\mathbf{X}-\mathbf{X} \mathbf{S}\parallel_{F}^{2} \\ & s.t.~ \mathbf{X}_{i,j}=\mathbf{M}_{i,j},\forall (i,j) \in N \end{aligned}  $$

Thus, each element in an incomplete matrix is represented by a linear combination of values of other elements in the matrix, the angles among these data points should be small enough, then the missing elements can be recovered with matrix completion based on sparse self-representation by solving the optimization problems shown in Eqs. (3) and (4).

The sparse self-representation based matrix completion is solved by applying *l*_1_ norm to the **S** in Eq. (3) as follow:

5 $$  \begin{aligned} \min\limits_{\mathbf{X},\mathbf{S}}\parallel \mathbf{S} \parallel_{1} ~~s.t.~ & \mathbf{X}=\mathbf{X} \mathbf{S},diag(\mathbf{S})=0,\\ & \mathbf{X}_{i,j}=\mathbf{M}_{i,j},\forall (i,j) \in N \end{aligned}  $$

By applying Frobenius norm to **S** in Eq. (4) and get

6 $$  \begin{aligned} \min\limits_{\mathbf{X},\mathbf{S}}& \parallel \mathbf{S} \parallel_{F}^{2}+\frac{\partial}{2} \parallel\mathbf{X}-\mathbf{X} \mathbf{S}\parallel_{F}^{2} \\ & s.t.~ diag(\mathbf{S})=0,\mathbf{X}_{i,j}=\mathbf{M}_{i,j},\forall (i,j) \in N \end{aligned}  $$

which is least-square self-representation based matrix completion. Setting the diagonal elements of **S** as zeros is to avoid that a data sample is reconstructed by itself.

### Learning an optimal kernel using unsupervised multiple kernel learning

#### Kernel function

Kernel functions define a non-linear mapping *Φ* from the input space $\mathcal {X}$ to a higher-dimensional Hilbert space $\mathcal {H}$. A function $k(\cdot,\cdot):\mathcal {X}\times \mathcal {X}\rightarrow \mathbb {R}$ is called a kernel over $\mathcal {X}$, denotes the similarity between input data samples *x* and *x*^′^. By applying a kernel function *k*(·,·) to any two data points $x,x^{\prime }\in \mathcal {X}$, *k*(*x*,*x*^′^) is equal to an inner product of mapping *Φ*(*x*) and *Φ*(*x*^′^) in the Hilbert space:

7 $$ \forall x,x^{\prime}\in\mathcal{X}, k\left(x,x^{\prime}\right)=\Phi (x)\cdot\Phi \left(x^{\prime}\right)  $$

For non-linear mapping $\Phi :\mathcal {X} \rightarrow \mathcal {H}$ to a Hilbert space $\mathcal {H}$ called a feature space. Since an inner product is a measure of the similarity of two vectors *Φ*(*x*) and *Φ*(*x*^′^), the kernel function *k*(·,·) is often interpreted as a similarity measure between points of the input space $\mathcal {X}$. An important advantage of a kernel function *k*(·,·) is efficiency: the computation of *k*(*x*,*x*^′^) is often significantly more efficient than the computation of an inner product of the non-linear mapping *Φ*(*x*) and *Φ*(*x*^′^) in Hilbert space $\mathcal {H}$.

#### Kernel learning method

Kernel learning methods embed the input data into a Hilbert space by specifying the inner product between each pair of data points. They are formulated as convex optimization problems, which have a single global optimum and do not require heuristic choices of learning rates, starting configurations or other parameters.

Let $\lbrace x_{i}\rbrace _{i=1}^{n}\subseteq \mathcal {X}$ be a collection of *n* data samples, and $\lbrace \Phi (x_{i})\rbrace _{i=1}^{n}\subseteq \Phi (\mathcal {X})$ be a non-linear mapping from the input space $\mathcal {X}$ to its corresponding Hilbert space $\mathcal {H}$, the function $k\left (x,x^{\prime }\right)=\Phi (x)\cdot \Phi \left (x^{\prime }\right)$ is called a kernel *k*(·,·). A kernel matrix $\mathbf {K}=(\mathbf {K}_{ij})^{n}_{i,j=1}\in \mathbb {R}^{n\times n}$ is a square matrix, where **K**_*ij*_=*k*(*x*_*i*_,*x*_*j*_) for the input data points $x_{1},\ldots,x_{n}\in \mathcal {X}$ and the kernel function *k*(·,·). The kernel matrix stores the inner product of features in the Hilbert space $\mathcal {H}$ so that it is constrained by:

8 $$ \sum\limits_{i} \Phi (x_{i})=0   $$

For the linear constraint on the elements of the kernel matrix, *Eq*. (8) can be rewritten in terms of the kernel matrix as follow:

9 $$ 0=\left\vert\sum\limits_{i} \Phi (x_{i})\right\vert^{2}=\sum\limits_{ij} \Phi (x_{i})\cdot\Phi (x_{j})=\sum\limits_{ij}\mathbf{K}_{ij}  $$

Thus, the kernel matrix is a symmetric positive semi-definite matrix that contains its entries the inner products between all pairs of input data points $x_{i}\subseteq \mathcal {X}$, and it determines the relative positions of those data points in the Hilbert space $\mathcal {H}$.

#### Unsupervised multiple kernel learning

Multiple Kernel Learning(MKL) methods [30] aim at learning a linear combination of a set of predefined basis kernels to identify an optimal kernel for the corresponding applications. Compared with conventional kernel methods only using a single predefined kernel function, MKL methods have the advantages of automatic kernel parameter tuning and capability of concatenating heterogeneous data. To choose the most suitable kernel and exploit heterogeneous features of input datasets, MKL methods construct a few candidate kernels and merges them to form a consensus kernel [26]. The traditional MKL algorithms are supervised learning since that the optimal kernel learning task requires the class labels of training data samples. However, the class labels of training data samples may not always be available prior to execute the MKL task in some real-world scenarios, such as clustering and dimension reduction. Unsupervised Multiple Kernel Learning(UMKL) determines a linear combination of multiple basis kernels by learning from unlabeled data samples, and the generated kernel can be used in data mining, such as clustering and classifying, as it is supposed to provide an integrated feature of input datasets [31]. Thus, to apply multiple kernels to clustering, MKDCI obtain an optimal kernel by the UMKL method.

Consider a set of *n* training data samples $\mathcal {D}=\lbrace (x_{1},y_{1}),\ldots,(x_{n},y_{n})\rbrace $, where $x_{i} \in \mathbb {R}^{d}$ is the feature vector of input data samples with *d* attributes, *y*_*i*_ is the unknown class label of the input data sample *x*_*i*_, {*k*_*t*_(·,·),*t*=1,…,*m*} is a set of *m* predefined basis kernel functions, and $\mathcal {K}_{conv}$ is the optimization domain of these candidate basis kernels including Gaussian kernel, Exponential kernel, Laplace kernel, etc. The goal of UMKL is to find an optimal linear combination of the *m* basis kernel functions, i.e., $ k_{w}(\cdot,\cdot)\in \mathcal {K}_{conv}$, and $\mathcal {K}_{conv}$ is defined as:

10 $$ \begin{aligned} \mathcal{K}_{conv}= &\left\{ k(\cdot,\cdot) = \sum\limits_{t=1}^{m} \mu_{t}k_{t}(\cdot,\cdot),\right.\\ &\left. \sum\limits_{t=1}^{m} \mu_{t}=1,\mu_{t}\geq 0\right\} \end{aligned}   $$

where each candidate kernel *k*(·,·) is the combination of *m* basis kernels {*k*_1_,…,*k*_*m*_}, *μ*_*t*_ is the coefficient(weight) of the *t*-th base kernel.

A simple choice for the coefficients *μ*_*t*_ is to set them all equal to 1/*m* regardless of the input dataset feature. However, this choice treats all the basis kernels identically and does not take into account the fact that some of the basis kernels can be redundant or atypical. The better choice is to solve an optimization problem so as to get a more suitably combined kernel for integrating all information of the input dataset. Based on the above definition of $\mathcal {K}_{conv}$, the key task of UMKL is to obtain an optimal kernel *k*(·,·) for the input dataset according to the unlabeled training data samples. Thus, the UMKL task can be formulated by utilizing the following optimization principles:

1. A suitably combined kernel enables each training data sample to be reconstructed from the localized bases weighted by the kernel values, i.e., for each data sample *x*_*i*_, the optimal kernel minimizes the approximation error $\left \| x_{i} - {\sum \nolimits }_{j} x_{j}k(x_{i},x_{j}) \right \|$.
2. An idea kernel induces the kernel values that are coincided with the original topology of the unlabeled training dataset, i.e., the optimal kernel minimizes the distortion over all training data samples ${\sum \nolimits }_{ij}k(x_{i},x_{j})\parallel x_{i} - x_{j} \parallel ^{2}$.

Besides, a set of local bases $\mathcal {B}_{i}$ for each sample *x*_*i*_ is introduced to infer a local structure, which is used to reconstruct data sample *x*_*i*_ and compute its distortion. According to the above two principles, the task of finding an optimally combined kernel with UMKL illustrated in Eq. (10) can be formulated as follows:

11 $$ \begin{aligned}  \min\limits_{k \in \mathcal{K}_{conv},\mathcal{B}}&\frac{1}{2}\sum\limits_{i=1}^{n} \left\| x_{i} - \sum\limits_{x_{j}\in \mathcal{B}_{i}} k_{ij}x_{j} \right\|^{2} \\ & + \gamma_{1}\sum\limits_{i=1}^{n}\sum\limits_{x_{j}\in \mathcal{B}_{i}}k_{ij} \parallel x_{i} - x_{j} \parallel^{2} + \gamma_{2}|\mathcal{B}_{i}| \end{aligned}  $$

where *k*_*ij*_=*k*(*x*_*i*_,*x*_*j*_), the target kernel *k* and local bases set $\mathcal {B}_{i}$ will be optimized by UMKL, the parameter *γ*_1_ controls the trade-off between the coding error and the locality distortion, and *γ*_2_ controls the size of local basis set $\mathcal {B}_{i}$.

To simplify the formulation (11), a matrix **D**∈{0,1}^*n*×*n*^ is introduced for each data sample *x*_*i*_, where each column vector *d*∈{0,1}^*n*^ indicate its neighbors, i.e., $\mathcal {B}_{i}=\lbrace x_{j}:d_{j}\neq 0\rbrace $. Besides, by constraining the size of each local base to certain constant, the optimization problem can be further rewritten as follows:

12 $$ \begin{aligned} \min\limits_{\mu \in \mathbf{\Delta},\mathbf{D}} &\frac{1}{2} \parallel \mathbf{X}(\mathbf{I}-\mathbf{K}\circ \mathbf{D}) \parallel^{2}_{F} + \gamma_{1} tr\mathbf{K}\circ \mathbf{D}\circ \mathbf{M}(\mathbf{1}\mathbf{1}^{\intercal}) \\ s.t. ~ ~&\mathbf{D}\in \lbrace 0,1\rbrace^{n\times n},\parallel \mathbf{d}_{i}\parallel_{1} =B,i=1,\ldots,n \\ & \mathbf{\Delta}=\left\{ \mathbf{\mu}:\mathbf{\mu}^{\intercal} \mathbf{1}=1,\mathbf{\mu}\geq0\right\}  \end{aligned}  $$

where the optimal kernel matrix **K** is determined by $[\mathbf {K}]_{ij}= {\sum \nolimits }_{t=1}^{m} \mu _{t}k_{t}(x_{i},x_{j}), 1\leq i,j\leq n$, *B*≤*n* denotes the size of $\mathcal {B}_{i}$ for each data samples *x*_*i*_, ∘ denotes an element-wise multiplication of two matrices, ∥·∥_*F*_ denotes the Frobenius-norm of a matrix, and *tr* denotes the trace of a matrix.

To apply the UMKL method, the input dataset is split into a training set and a test set with the ratio of 70:30 by randomly sampling, i.e., they account for 70% and 30% of entire input dataset respectively. According to each predefined basis kernel, *m* kernel matrices are computed for the training data samples, the parameters *γ*_1_ and *B* are estimated by cross-validation on the training data samples, and the above optimization problem can be solved with the algorithm discussed in [31]. Thus, by training on the unlabeled input dataset with the UMKL method, an optimally combined kernel *k*(·,·) with the weights of the predefined basis kernels *μ*_*t*_ are learned. The learned optimal kernel can be utilized to compute the local density of each data sample in the input dataset and dimensionality reduction of high-dimensional datasets.

### Computation of the optimal parameters

**Fig. 1:**
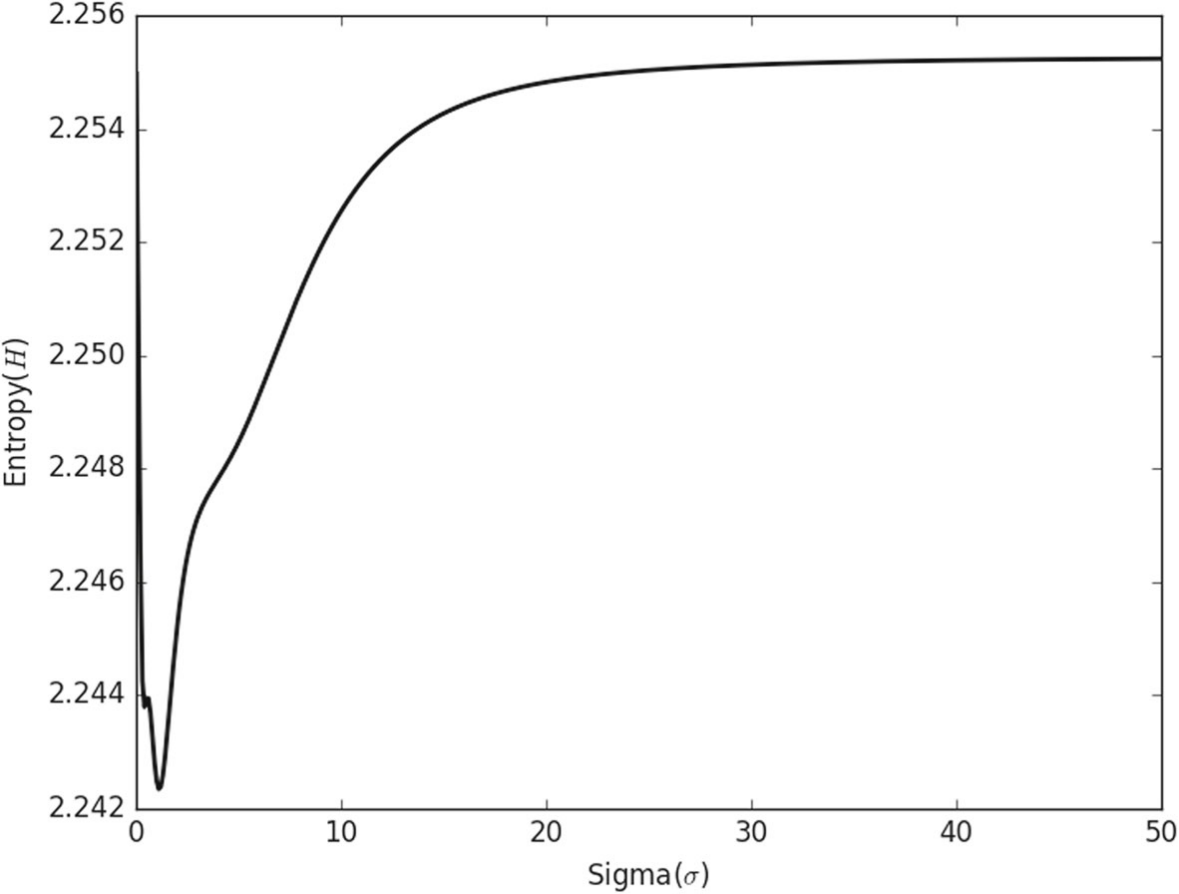
Distribution of entropy *H* with the different values of *σ* for the DLBCL-B dataset

According to the filed theory, if a data sample is treated as a physical object of the data field to diffuse its contribution on the clustering task, the potential value of an object *x*_*i*_ in a data field is:

13 $$ \varnothing(x_{i}) = \sum\limits_{j=1}^{n}\sum\limits_{t=1}^{m}\mu_{t}k_{t}(x_{i},x_{j})  $$

where *k*(·,·) is the kernel function learned by the UMKL method and defines the rule that how an object diffuses its contribution in the data field. The uncertainty of potential distribution is usually measured by the entropy *H* for the input dataset *X*^*n*×*d*^ defined as following:

14 $$ H = -\Sigma_{i}^{n} \frac{\varnothing_{i}}{Z} \log\left(\frac{\varnothing_{i}}{Z} \right), 0\leq H \leq \log(n)  $$

where $\varnothing _{i}$ is the potential value of each data point *x*_*i*_ in the scalar field, $Z={\sum \nolimits }_{i}^{n}\varnothing _{i}$ is a normalization factor. Since that the kernel *k*(·,·) is learned based on Gaussian kernel, Exponential kernel, Laplace kernel, etc., the values of entropy *H* change with different *σ* for the input dataset shown in Fig. 1. The value of *H* decreases quickly at first, then increases slowly and finally maintains the similar level when the parameter *σ* of basis kernels increases from 0 to *∞*.

The radius of attenuation is regarded as the impact scope for the optimal kernel function *k*(·,·), the value of the cut-off distance threshold *d*_*c*_ is determined by the radius of attenuation since that one data point only influence the other data points inside its radius. The most data points stochastically distribute inside the interval between the expectation plus threefold variances and the expectation minus threefold variances in a normal distribution [32], the radius of attenuation is $\frac {3\sigma }{\sqrt {2}}$ for each point of the data filed. Thus, the parameter *σ* obtained when the entropy *H* reaches the smallest value, and $\frac {3\sigma }{\sqrt {2}}$ is chosen as the optimal cut-off distance threshold *d*_*c*_ in the proposed MKDCI algorithm.

### Dimensionality reduction of input data samples

Data samples in the Bioinformatics datasets usually contain a lager number of attributes, and several applications require a clustering algorithm that can properly treat this type of large-scale high-dimensional datasets in terms of effectiveness and efficiency. To deal with these limitations and enable the proposed algorithm to be applied on the high-dimensional dataset effectively, the high-dimensional data samples are first mapped into two-dimensional space, for finding a non-linear mapping between high-dimensional space and low-dimensional space. The t-Distributed Stochastic Neighbour Embedding (t-SNE) [33] is a popular method that creates a two-dimensional map of data samples with hundreds or even thousands of dimensions. By introducing optimally combined kernel function *k*(·,·) to t-SNE, called Multiple Kernel t-SNE (MKt-SNE), dissimilarity of a pairwise data samples in high-dimensional space is defined as joint probabilities *p*_*ij*_ illustrated as Eq. (15), and the input matrix consists of distance between each pair data samples in the given dataset:

15 $$  \begin{aligned} & p_{ij} = \frac{p_{j|i} + p_{i|j}}{2n}, \\ & p_{j|i} = \frac{k(x_{i},x_{j})}{\Sigma_{t\neq i}k(x_{i},x_{t})}, p_{j|i} = 0 \end{aligned}  $$

where *k*(·,·) is the optimally combined kernel function for the input dataset which is obtained by the UMKL method.

In the low-dimensional space, the dissimilarity *q*_*ij*_ between two data samples *y*_*i*_ and *y*_*j*_ is measured by a normalized Student-t kernel is shown in Eq. (16):

16 $$  q_{ij} = \frac{\left(1+\parallel y_{i} - y_{j} \parallel^{2}\right)^{-1}}{\Sigma_{k\neq l}\left(1+\parallel y_{k} - y_{l} \parallel^{2}\right)^{-1}}, q_{ii} = 0  $$

The optimal locations of the data sample *y*_*j*_ are determined by minimizing the Kullback-Leibler divergence between the joint distributions *P* and *Q*:

17 $$ C(Y) = KL(P\parallel Q) = \Sigma_{i\neq j}p_{ij}\log\left(\frac{p_{ij}}{q_{ij}} \right)  $$

By minimizing *C*(*Y*) over all data samples in the input dataset, the objective function focuses on modeling similar objects with higher *p*_*ij*_ and its neighbor points with higher *q*_*ij*_ in two-dimensional space with the proposed MKt-SNE method. Due to the objective function is non-convex in low-dimension space, the objective function can be minimized by descending along the gradient:

18 $$ \begin{aligned} \frac{\partial C}{\partial y_{i}} & = 4\Sigma_{i\neq j}(p_{ij} - q_{ij})p_{ij}Z(y_{i} - y_{j}), \\ Z(y_{i}- y_{j}) & = \Sigma_{i\neq j} \left(1 + \parallel y_{i} - y_{j} \parallel^{2}\right)^{-1} \end{aligned}  $$

To approximate the MKt-SNE gradient, the gradient is split into two parts of *F*_*attr*_ and *F*_*rep*_, which denote the sum of all attractive forces and the sum of all repulsive forces respectively.

19 $$ \begin{aligned} \frac{\partial C}{\partial y_{i}} &= 4(F_{attr} + F_{rep})\\ &= 4\left(\Sigma_{i\neq j}p_{ij}q_{ij}Z(y_{i} - y_{j}) - \Sigma_{i\neq j}q^{2}_{ij}Z(y_{i} - y_{j}) \right) \end{aligned}  $$

Thus, a faithful representation in the two-dimensional space for each data sample in the input dataset can be found with the MKt-SNE method. The method preserves both local and global information of data samples in the corresponding low-dimensional space [34] and is suitable to be applied on the large-scale datasets with several attributes.

### Calculation of local density and minimum distance

There are two critical parameters for each data samples *x*_*i*_ must be calculated for the proposed MKDCI algorithm, i.e., its local density *ρ*_*i*_ and minimum distance *δ*_*i*_ from other data samples with higher local density. Let the distance between each pair of data samples *x*_*i*_ and *x*_*j*_ be denoted as *d*(*x*_*i*_,*x*_*j*_), the local density *ρ*_*i*_ of a data sample *x*_*i*_ denotes the number of data samples that are closer than the cut-off distance threshold *d*_*c*_ to itself and is defined as:

20 $$ \rho_{i} = \sum\limits_{j=1}^{n} X(d(x_{i},x_{j}) - d_{c})  $$

The value of *ρ*_*i*_ is affected by statistical errors and the kernel function that maps the data samples into new vector spaces where the data samples become more easily separated or better structured, thus the optimally combined kernel functions base on multiple basis kernels can be carried into a new vector space without explicitly mapping the input data samples into this space. In the UMKL method, each data sample has multiple features representations by learning an optimally combined kernel, and the similarity between data samples can be estimated with the optimal kernel function. Thus, the local density *ρ*_*i*_ with the optimally combined kernel *k*(·,·) is estimated by Eq. (21), which utilizes the optimal cut-off distance *d*_*c*_ as the input parameter instead of the parameter *σ* in predefined basis kernel functions:

21 $$  \rho_{i} = \sum\limits_{j=1}^{n} k(x_{i}, x_{j}; d_{c})  $$

Correspondingly, the minimum distance between the data sample *x*_*i*_ and other data samples with higher local density denoted by *δ*_*i*_, is defined as:

22 $$  \delta_{i} = \left\{ \begin{aligned} \min\limits_{j:\rho_{j}>\rho_{i}}(d_{ij}), ~ \text{if} \exists\rho_{j} > \rho_{i} \\ \max\limits_{j}(d_{ij}), ~ \text{if} \nexists \rho_{j} > \rho_{i} \end{aligned}\right.  $$

### Estimation of cluster centroids

To detect the suitable cluster centroids is the critical step of the proposed MKDCI algorithm for generating the optimum clustering results. In the MKDCI algorithm, the cluster centroids are the set of data samples with higher local density *ρ*_*i*_ and larger relative distance *δ*_*i*_, the parameter *θ*_*i*_=*ρ*_*i*_×*δ*_*i*_ transforms the local density *ρ*_*i*_ and relative distance *δ*_*i*_ of each data sample into one parameter.

Since the outliers are few and different data samples in the dataset, outlier detection methods can be used to automatically detect cluster centroids based on the set of local density *ρ*_*i*_ and parameter *θ*_*i*_ in the MKDCI algorithm. Thus, cluster centroids with lager *θ*_*i*_ will be automatically detected by searching for outliers in the set of variable *θ*_*i*_ with the outlier detection method. Nevertheless, the data samples both with high *ρ*_*i*_ and low *δ*_*i*_, and with low *ρ*_*i*_ and high *δ*_*i*_ will be assigned with high *θ*_*i*_. Thus false cluster centroids may be generated when the set of variable *θ*_*i*_ is only searched. Therefore, the outliers in the set of variable *δ*_*i*_ are also searched with the outlier detection method. Then, the potential cluster centroids are determined by the intersection of the two sets of outliers detected from both *θ*_*i*_ and *δ*_*i*_.

**Fig. 2:**
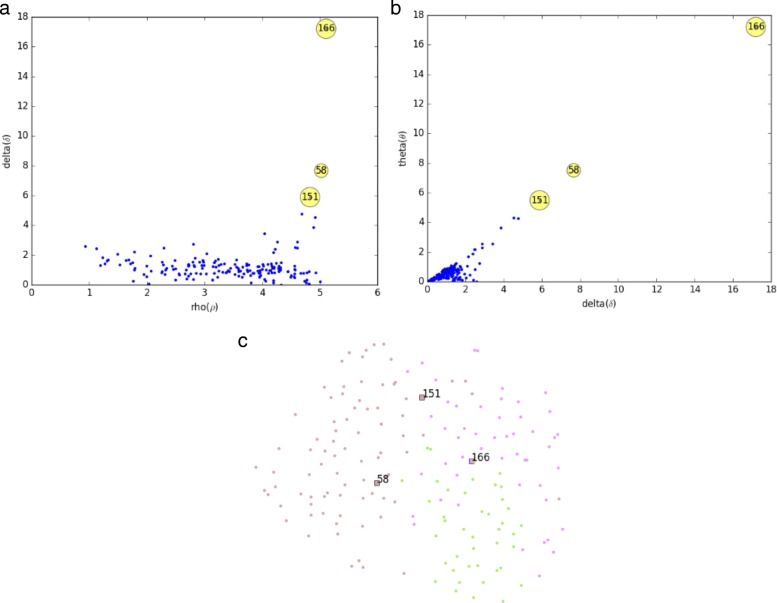
Illustration of potential cluster centroids automatically detected using the proposed MKDCI algorithm on the DLBCL-B dataset. **a** Scatter plot of the distribution of potential cluster centroids in which plots of delta(*δ*) against the rank of rho(*ρ*), and potential cluster centroids have significantly higher values of *δ* and *ρ*. **b** Scatter plot of the distribution of potential cluster centroids in which plots of delta(*δ*) against theta(*θ*) are generated with MKDCI’s automatic detection method, and potential cluster centroids are easier recognized in this region. **c** Scatter plot of the DLBCL-B dataset with three ground-truth clusters, ground-truth clusters are color labeled, and potential cluster centroids are labeled by its data point index with a square

There are several outlier detection methods, such as Grubbs test, Dixon test, generalized Extreme Studentized Deviate (ESD) test [35], Isolation Forests [11], etc. Although generalized ESD test is much better than Grubbs and Dixon test, it has the limitations that the distribution of the univariate data samples approximately follows a normal distribution and the number of data samples should be larger than 25. Isolation Forests method detects outliers in the set of univariate data samples regardless of the size of the dataset, and it detects outliers purely based on the concept of isolation without employing any distance or density measure, i.e., fundamentally different from other existing methods. It isolates samples by randomly selecting a feature and then randomly selecting a split value between the maximum and minimum values of the selected feature. To isolate anomaly samples are easier as only a few conditions are needed to separate those cases from the normal samples. Therefore, the Isolation Forest algorithm constructs the separation by firstly creating random decision trees. Then, the anomaly score is calculated as the path length to isolate the given dataset. To avoid issues caused by the randomness of the decision tree algorithm, the process is repeated several times, and the average path length is calculated and normalized. Moreover, this method has the low linear time complexity and a small memory requirement, and is more effective and efficient than other ones using distance and density measures. Therefore, the Isolation Forests method is more suitable to detect the potential cluster centroids in the MKDCI algorithm automatically. Figures 2a and b show the distribution of potential cluster centroids for the DLBCL-B bioinformatics dataset. It is found that the delta (*δ*) against the rank of theta (*θ*) is more suitable than delta (*δ*) against the rank of rho (*ρ*) to detect potential cluster centroids in the dataset. Figure 2c is the visualization of the ground-truth clusters and potential cluster centroids for the DLBCL-B bioinformatics dataset with the MKt-SNE method.

There might be multiple potential cluster centroids that have short relative distance between each other. Thus, the false cluster centroids should be deleted. First, the potential cluster centroids are sorted in descending order according to their local density, and the first cluster centroid is considered as the first actual cluster centroid. If the minimum distance between another potential cluster centroid and the known actual cluster centroids is shorter than the cut-off distance threshold *d*_*c*_, the potential cluster center will be removed from the set of potential cluster centroids, and become a member of the cluster. Otherwise, the potential cluster centroid is recognized as a new actual cluster center to form another cluster. Finally, the actual cluster centroids will be generated by refining those potential cluster centroids.

### Assignment of data samples

For the proposed MKDCI algorithm, the last step is to assign the remaining data samples to the corresponding cluster according to its both *ρ*_*i*_ and *δ*_*i*_ of the nearest neighbors. First, according to detected cluster centroids, the remaining data samples are assigned to its nearest cluster centroids with higher *ρ*_*i*_ as follows:

23 $$ D_{k} = \left\{ \begin{aligned} x_{i}, & \text{ if}\ i \in \text{centroids}\\ x_{j}, & \text{ otherwise,}\ \rho_{j} > \rho_{i} \wedge d_{ij} < d_{c} \end{aligned}\right.  $$

Second, to recognize the noise points, a border region for each cluster is defined as the set of data samples assigned to the cluster *D*_*k*_, but being within the cut-off distance threshold *d*_*c*_ from data samples assigned to other clusters *D*_*k*:*k*≠*l*_. For the cluster *D*_*k*_, the MKDCI algorithm searches the lowest density *ρ*_*b*_ within its border region, the data samples with a local density higher than *ρ*_*b*_ and belonging to the cluster *D*_*k*_ are assigned as the data samples of this cluster. The other data samples in the cluster *D*_*k*_ are determined as noise. Thus, the assignment of data samples is completed only in a single step, in contrast with other clustering algorithms where the generation of correct clusters usually needs to be optimized iteratively.

**Fig. 3:**
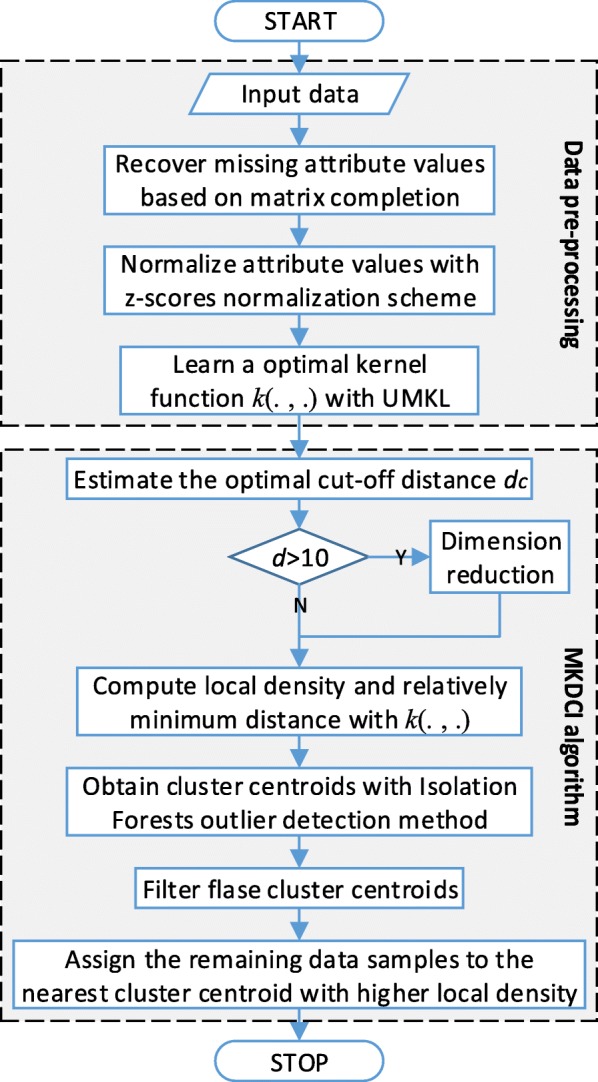
Flowchart of MKDCI algorithm

Thus, the flowchart of MKDCI algorithm is concluded in Fig. 3. To improve the performance of density clustering with proposed MKDCI algorithm, datasets should be pre-processed, such as to recover the missing attribute values of data samples and to normalize attribute values.

## Results

In this section, test datasets and corresponding pre-processing methodology are described firstly. Then, the implementation trick of MKDCI algorithm are explored, i.e., the input distance matrix is calculated by the split-apply-combine strategy so that the proposed clustering algorithm can efficiently process high-dimensional datasets with millions of data samples. Finally, the evaluation metrics, extensive experiments and their results are discussed in detail.

### Datasets and pre-processing

To evaluate the quality of the proposed MKDCI algorithm, the experiments on the following well-known bioinformatics datasets have been carried out:

1. Primary Biliary Cirrhosis (PBC) dataset contains the follow-up laboratory data for each studied patient with fatal chronic liver disease of unknown cause. Between 1974 and 1984, a double-blinded randomized clinical trial conducted in primary biliary cirrhosis of the liver, recording a large number of clinical, biochemical, serologic, and histologic parameters. This dataset also records the survival status of these studied patient in 1986.
2. Anuran Calls (MFCCs) dataset is used to recognize anuran species through their calls. It is a multi-label dataset with three labels, and the records belong to 4 different families, 8 genera, and 10 species according to 7195 syllables. In the following experiments, the species labels severed as the ground truth labels.
3. Diffuse large B-cell lymphoma (DLBCL-B) dataset contains the data samples deriving from germinal center cells, which can be distinguished from their immunoglobulin gene rearrangements, morphologic, molecular characteristics and clinical presentation. Disease staging and choice of treatment, including the type, number, sequence of chemotherapy agents and the need for consolidative radiation therapy, should be made base on these clinical factors, which collectively determine response to therapy and survival.
4. The other four bioinformatics datasets derive from UCI Machine Learning Repository (http://archive.ics.uci.edu/ml) including Wine, Breast Cancer Wisconsin Diagnostic (WDBC), Mice Protein Expression (MPE) and Epileptic Seizure Recognition (ESR) dataset. Wine dataset contains the results of a chemical analysis of wines grown in the same region but derived from three different cultivars, and the analysis determines the quantities of 13 constituents found in each type of wines. WDBC dataset consists of features which were computed from digitized images of FNA tests on a breast mass. MPE dataset consists of the expression levels of proteins/protein modifications that produced detectable signals in the nuclear fraction of the cortex. ESR dataset is a pre-processed and re-structured/reshaped version of a very commonly used dataset featuring epileptic seizure detection.

First, attributes with missing values in datasets will result in returning with error values during the process of clustering. For instance, PBC dataset contains 72 incomplete data samples that account for 20.52% of all data samples, they comprise 128 missing values in total. MPE dataset contains 528 incomplete data samples that account for 48.89% of all data samples, they comprise 1396 missing values in total. To compare with the traditional method of filling missing values, preprocessed datasets PBC and MPE are denoted as PBC-A and MPE-A respectively when missing attribute values of data samples are filled with the average value of corresponding attributes. Otherwise, preprocessed datasets PBC and MPE are denoted as PBC-R and MPE-R respectively when missing attribute values are recovered with the method of matrix completion based on spare self-representation. Second, since the high-dimensional dataset contains several attribute values of varying scale, these attribute values of data samples in the training set and the test set are normalized with z-scores normalization scheme shown in Eq. (24), to avoid inappropriate assignment of data samples during clustering.

24 $$  Z = \frac{X-\mu}{\sigma}  $$

where *X* is values of one attribute for each data sample in the input dataset to be normalized, *μ* is the mean value of this attribute, and *δ* is the standard deviation of this attribute. After pre-processing with above two steps, the selected datasets are described in Table 1.

**Table 1:** Summarizes the properties of the datasets

Properties	PBC-A	PBC-R	MFCCs	DLBCL-B	Wine	WDBC	MPE-A	MPE-R	ESR
*k*	4	4	10	3	3	3	8	8	5
*dim*	18	18	22	643	13	30	80	80	178
*N*	624	624	7195	180	178	569	1080	1080	11500

*k* is the number of ground-truth clusters in each dataset, *dim* is the dimension of each data sample in the datasets, and *N* is the number of data samples in the datasets

### Computation of distance matrix for large-scale datasets

To compute and store the entire distance matrix for a large-scale dataset with millions of data samples is memory intensive, and the matrix tends to beyond the memory capacity of current personal computers. The split-apply-combine strategy [36] breaks up a big matrix into manageable chunks, operate on each chunk independently and then pulls the chunks together. Thus, the proposed MKDCI algorithm utilizes the split-apply-combine strategy to calculate and store the distance matrix, in order to make the algorithm applicable to the large-scale bioinformatics datasets.

**Fig. 4:**
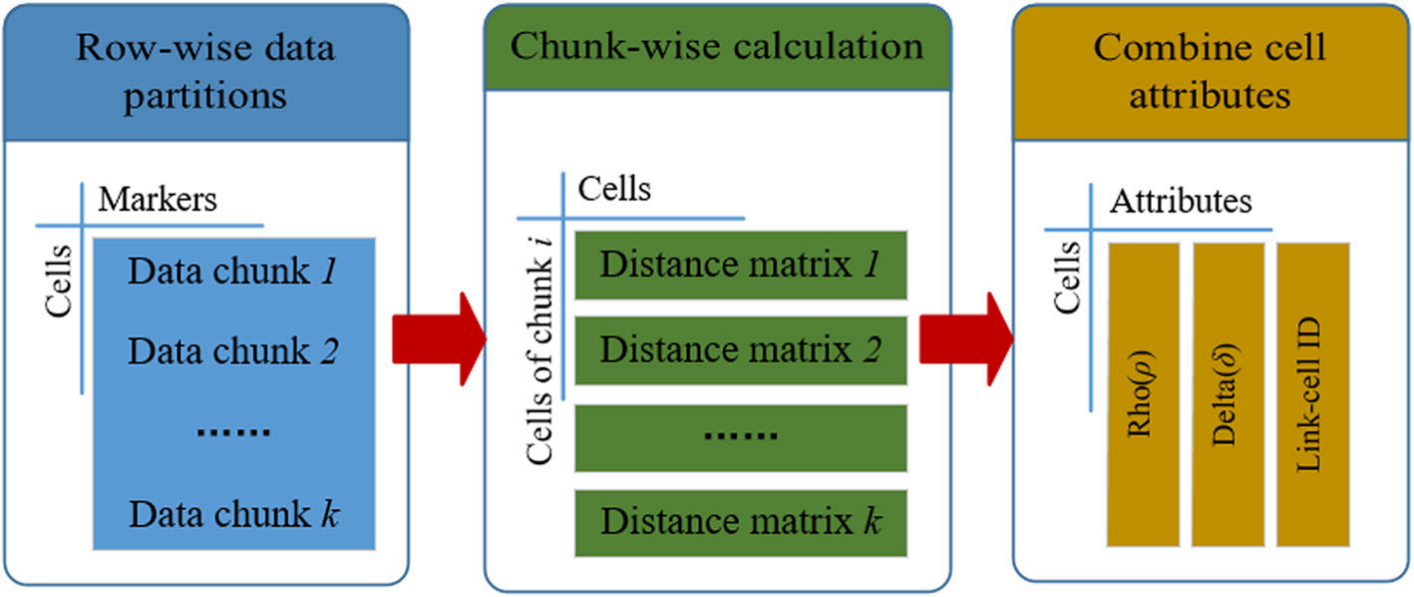
Split-apply-combine strategy in MKDCI

As illustrated in Fig. 4, the data samples in the given dataset firstly are split row-wisely into *k* different chunks, then the corresponding distance matrix for each chunk is computed independently, in order to restrict the distance matrix in a limited size. Besides the local density *ρ*_*i*_ and relatively minimum distance *δ*_*i*_, and a new parameter called link-cell *ID* is calculated for each chunk, which is the index of its nearest point with higher density to avoid duplicate calculations in the final assigning step of the proposed MKDCI algorithm. Finally, these parameters from all chunks are combined to automatically detect cluster centroids and assign remaining data samples to correct clusters. Thus, the split-apply-combine strategy implied in the MKDCI algorithm reduces the memory burden and also calculates distance matrix to be accelerated on multi-core CPUs and many-core GPUs [37–39].

### Evaluation metrics of clustering quality

There are mainly three types of clustering evaluation metrics that are widely used, namely contingency table-based measures, pairwise measures and entropy-based measures. Contingency table-based measures, such as accuracy, error rate and *F-measure (F-m)*, assume that the ground-truth clustering labels are known as a priori. Pairwise measures, such as *Adjusted Rand-Index (ARI)* and *Adjusted Rand Error (aRe)*, utilize the partition information and the clustering labels over all pairs of data samples. Entropy-based measures, such as *Adjusted Mutual Information (AMI)* and *Normalized Mutual Information (NMI)*, make use of entropy concept as well as ground-truth clustering labels to evaluate the clustering results.

*F-m* and *aRe* are not suitable to describe a comparison among different clustering algorithms on the datasets with numerous noisy data samples because they only take already clustered data samples into account. Hence, *AMI* and *NMI* are employed to quantify the amount of shared information between the clusters obtained by the clustering algorithm and the given ground-truth clusters in the datasets. Thus, four different metrics are jointly used in this section to evaluate the quality of different clustering algorithms, including *F-m*, *aRe*, *NMI* and *AMI*.

To compute the metrics of clustering evaluation, assume the set *C* is the distribution of the ground-truth clustering labels in the input dataset, which contains *n* data samples and is partitioned into *t* subsets {*C*_1_,…,*C*_*t*_}. Meanwhile, the distribution of clustering results is the set *D*={*D*_1_,…,*D*_*k*_}, which is obtained by a clustering algorithm applied to the same dataset.

#### *F-m* and *aRe*

Accuracy can be ambiguous, because it only evaluates the exactness of individual clusters, regardless of the overall number of clusters. Thus the larger the number of identified clusters is, the higher the accuracy will be. Meanwhile, error rate only calculates the mispredicted ratio of individual clusters, regardless of the total number of clusters, leading to the clusters with more mispredicted samples have the higher error rate. Whereas, *F-m* takes the overall number of clusters into account and keeps a balance between the overall number of clusters and the accuracy (or error rate) of individual clusters.

The *F-m* measures the success of retrieving the ground-truth clusters *C* in items of the precision and recall of clustering results *D* produced by the clustering algorithm, whereby the prefect clustering result is denoted by *F-m*=1.

Let the parameters be denoted as follows:

- *m*_*i*,*j*_=|*C*_*i*_∩*D*_*j*_| is the number of data samples in the ground-truth cluster *C*_*i*_ assigned to the generated cluster *D*_*j*_ by a clustering algorithm,
- *m*_*i*,*all*_=|*C*_*i*_| is the total number of data samples in the ground-truth cluster *C*_*i*_,
- *m*_*all*,*j*_=|*D*_*j*_| is the total number of data samples in the generated cluster *D*_*j*_,
- *m*_*all*,*all*_=|*C*| is the total number of data samples in the dataset except the data samples that are difficult to clustering, i.e., the values of their ground-truth clustering labels are -1.

Thus, for each pairwise cluster *C*_*i*_ and *D*_*j*_, $precision(i,j)= \frac {m_{ij}}{m_{all,j}}$ and $recall(i,j)=\frac {m_{ij}}{m_{i,all}}$ are computed, and *F-m* is defined as:

25 $$ \begin{aligned} &{F-m} = \sum\limits_{i}^{t}\frac{m_{i,all}}{m_{all.all}}\times \max_{j}\{ F(i,j) \},\\ &F(i,j) = 2\times \frac{precision(i,j)\times recall(i,j)}{precision(i,j)+ recall(i,j)} \end{aligned}  $$

*aRe* is derived from the *ARI* and measures the differences between the ground-truth clusters and clustering results produced by a clustering algorithm. *ARI* measures the concordance between different clustering results, and is defined as:

26 $$ \begin{aligned} & ARI = \frac{{n \choose 2}(m_{i,j}+m_{all,j}) - (u+v) }{{n \choose 2}^{2} - (u+v)},\\ & u=\left(m_{i,j}+m_{all.all}\right)\left(m_{i,j}+m_{i,all}\right),\\ & v = \left(m_{i,all}+m_{all,j}\right)\left(m_{all,all}+m_{all,j}\right) \end{aligned}  $$

The perfect clustering algorithm is that the predicted clusters generated by the algorithm are identical to the ground-truth clusters. Thus, *aRe* is defined as 1−*ARI*, the prefect clustering clusters is denoted by *aRe*=0.

#### *NMI* and *AMI*

By comparing clustering results with corresponding ground-truth clusters directly based on the data samples, it is hard to decide whether the assignment of one clustering result is right or wrong for the given dataset. Therefore, an effective method to evaluate the quality of the clustering results is to measure the relationships of each pair of data samples in the dataset. For each pair of data samples that share at least one cluster in the overlapping clustering results, pairwise measures try to estimate whether the prediction of this pair as being in the same cluster was correct with respect to the true underlying categories in the dataset.

*NMI* evaluates the similarity between the ground-truth labels of data samples and the clustering results in an information theoretic sense that makes a trade-off between the number of clusters and quality. It is computed by regarding the ground-truth labels and clustering results as the random variable *X* and *Y* respectively, and is formulated as [40]:

27 $$ {NMI}(X,Y) = \frac{I(X,Y)}{\sqrt{H(X)H(Y)}}  $$

Specifically, *AMI* is a variation of mutual information and corrects the effect of agreement solely due to the changes between two clusters, which is defined as Eq. (28):

28 $$  {\begin{aligned} {AMI}(X,Y) &= \frac{I(X,Y)-E(I(X,Y))}{\max\{ H(X),H(Y) \} - E(I(X,Y))} \\ I(X,Y) &= \sum\limits_{i=0}^{t-1}\sum\limits_{j=0}^{k-1}N_{ij}\log\left(\frac{{nN}_{ij}}{N_{i}N_{j}} \right), \\ H(X) &= \sum\limits_{i=0}^{t-1}N_{i}\log\frac{N_{i}}{n},\\ H(Y) &= \sum\limits_{j=0}^{k-1}N_{j}\log\frac{N_{j}}{n} \end{aligned}}  $$

where *I*(*X*,*Y*) is the mutual information between the ground-truth labels *X* and clustering results *Y*, it is a non-negative quantity upper bounded by the entropies *H*(*X*) and *H*(*Y*). *H*(*X*) and *H*(*Y*) are the entropy of *X* and *Y* respectively, max{*H*(*X*),*H*(*Y*)} denotes the maximum entropy of *X* and *Y*, and *E*(*I*(*X*,*Y*)) is the expected value of *I*(*X*,*Y*). *N*_*ij*_ denotes the number of data samples belonging to both cluster *C*_*i*_ and *D*_*j*_, *N*_*i*_ and *N*_*j*_ denote the number of data samples in the cluster *C*_*i*_ and *D*_*j*_ respectively. The range of *NMI* and *AMI* is from 0 to 1. Their values are larger denotes that the clustering results are better, and the value equal to 1 denotes that the two clusters are identical.

### Evaluation results

To evaluate the performance of the proposed MKDCI algorithm on the seven bioinformatics clustering datasets shown in Table 1, the selected basis kernels contain the Gaussian kernel, Exponential kernel, and Laplace kernel. Compared with three existing well-known density-based clustering algorithms, namely DBSCAN, HDBSCAN, DENCLUE2.0, and a parameter-free clustering algorithm PFClust, the quality evaluation results on the seven bioinformatics clustering datasets are illustrated in Table 2.

**Table 2:** Quality comparison of different clustering algorithms on bioinformatics datasets

Dataset	Measure metrics	PBC-A	PBC-R	MFCCs	DLBCL-B	Wine	WDBC	MPE-A	MPE-R	ESR
MKDCI	*F-m*	0.351	0.360	0.728	0.749	0.652	0.858	0.470	0.482	0.491
	*aRe*	0.956	0.953	0.406	0.526	0.704	0.382	0.693	0.689	0.852
	*NMI*	0.351	0.362	0.692	0.532	0.414	0.495	0.538	0.554	0.446
	*AMI*	0.070	0.076	0.615	0.496	0.379	0.453	0.429	0.438	0.219
DBSCAN (*MinPts*=4, *ε*_1_)	*F-m*	0.660	0.665	0.509	0.510	0.576	0.811	0.448	0.452	0.350
	*aRe*	0.999	0.998	0.858	0.956	0.772	0.602	0.796	0.794	0.967
	*NMI*	0.023	0.026	0.221	0.054	0.361	0.395	0.492	0.499	0.060
	*AMI*	0.005	0.005	0.124	0.039	0.269	0.295	0.347	0.347	0.003
HDBSCAN (*MinPts*=4)	*F-m*	0.623	0.627	0.785	0.565	0.620	0.853	0.265	0.271	0.332
	*aRe*	0.998	0.998	0.260	0.985	0.715	0.386	0.926	0.923	0.989
	*NMI*	0.029	0.032	0.686	0.174	0.386	0.469	0.518	0.523	0.082
	*AMI*	0.019	0.020	0.613	0.115	0.353	0.373	0.335	0.337	0.020
DENCLUE2.0 (*ε*_2_,*h*=*std*(*X*)/5)	*F-m*	0.023	0.025	0.415	0.493	0.372	0.007	0.304	0.308	0.650
	*aRe*	0.997	0.996	0.983	0.987	0.908	0.998	0.708	0.699	0.685
	*NMI*	0.344	0.347	0.105	0.184	0.385	0.322	0.472	0.478	0.472
	*AMI*	0.061	0.064	0.018	0.114	0.122	0.002	0.392	0.396	0.201
PFClust	*F-m*	0.315	0.320	0.375	0.442	0.373	0.432	0.202	0.207	0.271
	*aRe*	0.981	0.978	0.887	0.993	0.971	0.988	0.998	0.998	0.872
	*NMI*	0.002	0.002	0.123	0.043	0.033	0.019	0.024	0.028	0.135
	*AMI*	0.001	0.001	0.094	0.001	0.001	0.007	0.006	0.007	0.111
Parameters	*ε*_1_	24.657	24.657	0.306	19.819	3.626	20.413	2.221	2.221	1.426
	*ε*_2_	19.591	19.591	0.306	0.413	6.552	1.426	0.432	0.432	1.853

*MinPts* is the minimum number of data samples required to form a cluster, *ε*_1_ is the maximum distance between two data samples for them to be considered as in the same neighborhood, *ε*_2_ is the convergence threshold for density attractors and *h* is the parameter of a Gaussian kernel. *ε*_1_ and *ε*_2_ are the corresponding parameters when the better clustering results are obtained for *F-m* evaluation metric during clustering with ten random values of the parameters between 0.0 and 50.0

## Discussion

Compared with the PFClust algorithm, the proposed MKDCI algorithm significantly improves the quality of the parameter-free clustering. Meanwhile, MKDCI algorithm also automatically generate clustering results of higher quality on the most of high-dimensional bioinformatics datasets. The reason is that the utilized UMKL methods can obtain the optimal map between high-dimensional data samples and the low-dimensional data samples, and MKDCI algorithm automatically determines the optimally combined kernel function and similarity measure for dimensionality reduction and density clustering respectively. Compared with the method of filling missing attribute values of data samples with the average value, the method of matrix completion can improve slightly the performance of clustering algorithm. But the improvement of performance of MKDCI algorithm is mainly attributed to the optimization of combined kernels learned with UMKL. However, for the part of evaluation metrics on the PBC, MFCCs and ESR datasets, such as *F-m* and *aRe*, the quality of the clustering results generated by the MKDCI algorithm is lower than the ones generated by the HDBSCAN and DENCLUE2.0 algorithms. This is because these evaluation metrics only take already clustered data samples into account. The other four density clustering approaches need to determine parameters manually beforehand, and the clustering results heavily depend on the user’s experience, while the advantage of MKDCI algorithm is free from requiring determination of critical parameters by users. Thus, the proposed MKDCI is an efficient unsupervised learning algorithm. It is especially suitable for analyzing the high-dimensional bioinformatics data samples in a wide variety of applications, since that it aims to determine an optimally linear combination of multiple basis kernels by learning from the unlabeled dataset and automatically complete the clustering process without critical parameters determined manually by users in advance.

**Fig. 5:**
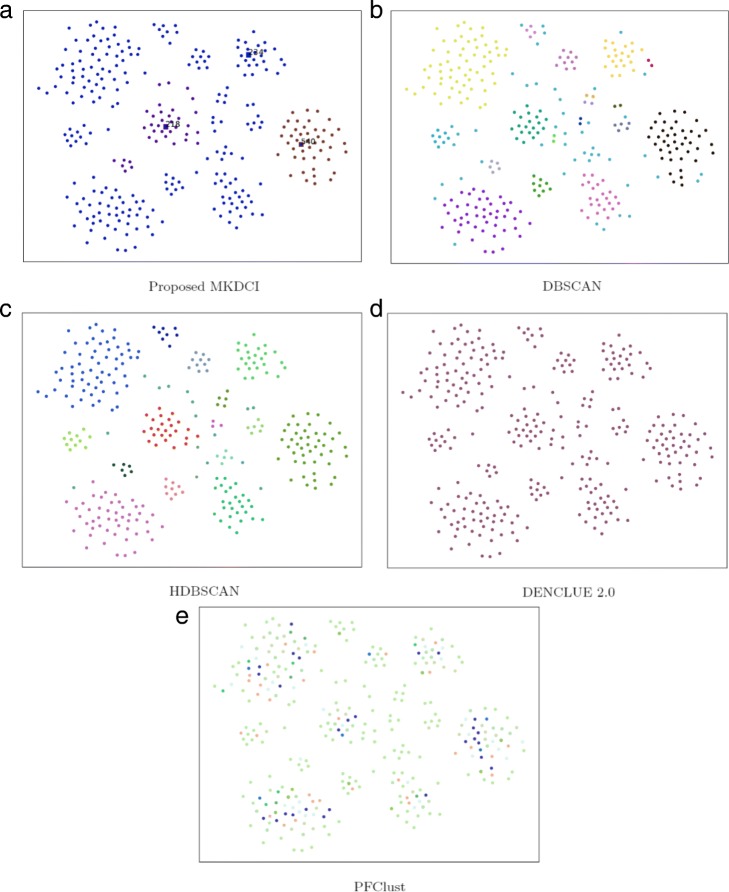
Illustration of results for the proposed MKDCI algorithm and other density clustering algorithms on PBC-R dataset. The numbers in **a** denotes cluster centroids obtained with the proposed MKDCI algorithm. **b**, **c**, **d** and **e** denote cluster results on PBC-R dataset generated by clustering algorithm DBSCAN, HDBSCAN, DENCLUE 2.0, PFClust respectively

**Fig. 6:**
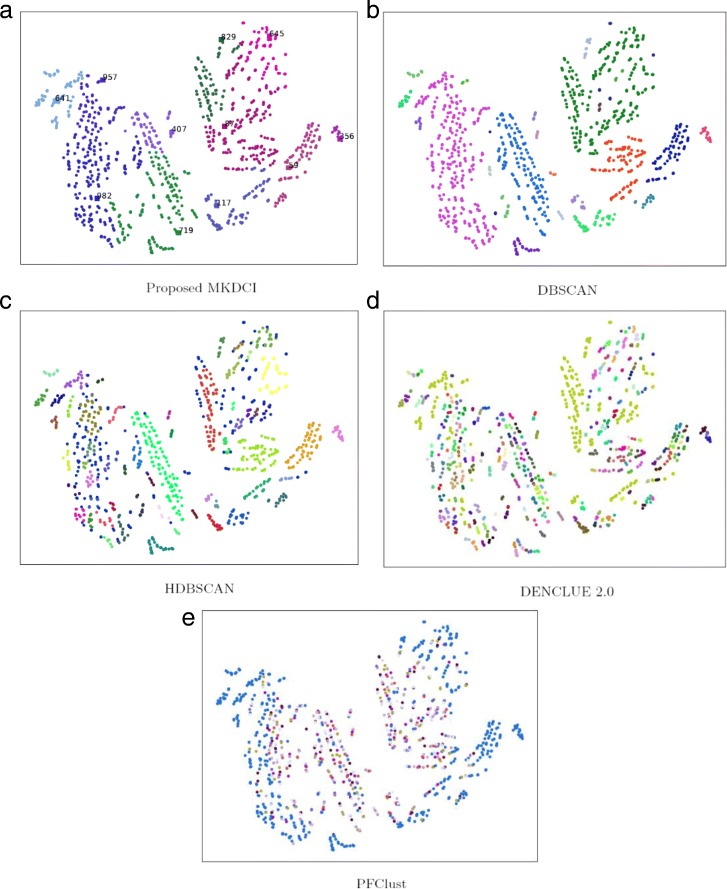
Illustration of results for the proposed MKDCI algorithm and other density clustering algorithms on MPE-R dataset. The numbers in **a** denotes cluster centroids obtained with the proposed MKDCI algorith. **b**, **c**, **d** and **e** denote cluster results on MPE-R dataset generated by clustering algorithm DBSCAN, HDBSCAN, DENCLUE 2.0, PFClust respectively

Meanwhile, to visualize the results of different clustering algorithms, 2D figures of t-SNE for the proposed MKDCI algorithm and other density clustering algorithms on PBC-R and MPE-R datasets are shown in Figs. 5 and 6.

## Conclusions

The proposed MKDCI algorithm provides an automatic density clustering approach with multiple kernels for bioinformatics datasets. It is especially suitable for larger-scale incomplete datasets in bioinformatics by combining the advantages of the density clustering method, prediction of the missing attribute values of data samples with the matrix completion method, the UMKL method for unlabeled training data samples, detection of cluster centroids based on the Isolation Forests method. The quality of the proposed MKDCI algorithm is evaluated with several well-known evaluation metrics, the results on multiple bioinformatics datasets with missing attribute values show that the MKDCI algorithm generates better clustering results than most of density clustering methods and the PFClust parameter-free clustering method. However, the optimal kernel used in the MKDCI algorithm is only the combination of three pre-specified basis kernels, the performance of the clustering can be improved by utilizing more basis kernels to obtain more suitable kernel function. Meanwhile, due to the sensitivity and privacy of the bioinformatics datasets, the privacy-preserving clustering method based on differential privacy is another promising topic for the future research.
